# Perceived Age Discrimination in the Second Half of Life: An Examination of Age, Period, and Cohort Effects

**DOI:** 10.1093/geroni/igad094

**Published:** 2023-08-30

**Authors:** Liat Ayalon, Octavio Bramajo

**Affiliations:** Louis and Gabi Weisfeld School of Social Work, Bar Ilan University, Ramat Gan, Israel; Centre D’Estudis Demogràfics, Autonomous University of Barcelona, Barcelona, Spain

**Keywords:** Age–period–cohort, Ageism, Discrimination, Subjective

## Abstract

**Background and Objectives:**

Ageism is defined as stereotypes, prejudice, and discrimination based on age. Perceived age discrimination (e.g., the behavioral component of ageism) is highly prevalent in society, as reported by 1 in 3 people in Europe. The present study examined variations in perceived age discrimination in the second half of life. We adopt a comprehensive approach that examines whether perceived age discrimination varies by age (chronological time from birth), period (the context when data were collected), or cohort (a group of people with shared life events experienced at a similar age) across gender and ethnic origin.

**Research Design and Methods:**

We relied on psychosocial data from the Health and Retirement Survey between 2006 and 2018. We ran a set of age–period–cohort models to determine the separate effects of aging (age) factors, contextual (period) factors, and generational (cohort) factors on perceived age discrimination.

**Results:**

Our findings show that perceived age discrimination increases with age but reaches a plateau around the age of 75. There also were some cohort effects, but they appeared minimal and inconsistent. No period effects were found.

**Discussion and Implications:**

The findings attest to the consistent nature of perceived age discrimination, which is less likely to be affected by external contextual events. It also is less likely to be affected by gender or ethnicity. The findings also suggest that it is older persons who are more likely to report age discrimination, thus, interventions should address ageism in this age group.

**Translational Significance:** We examined variations in perceived age discrimination (e.g., the behavioral component of ageism) in the second half of life. Age, rather than cohort or period, plays a consistent role in people’s reports of perceived age discrimination. This possibly attests to the consistent nature of perceived age discrimination, which is less likely to be affected by external contextual period or cohort effects. It also is less likely to be affected by gender or ethnicity. Intergenerational contact, education about ageism, and laws result in reduced levels of ageism and can possibly result in reduced age discrimination.

Ageism is defined as stereotypes, prejudice, and discrimination based on age (Ayalon & Tesch-Römer, 2018; Iversen et al., 2009). Based on the European Social Survey, one in three people in Europe reports exposure to ageism (e.g., perceived age discrimination; Ayalon, 2014) and one in two people reports being ageist (e.g., ageist stereotypes and prejudice), based on the World Value Survey (Officer et al., 2020). Ageism occurs at the macro, structural level; at the meso, interpersonal level; and at the micro, intrapersonal level (Ayalon & Tesch-Römer, 2018). It is manifested in all spheres of life, including the health care system, the workforce, the media, the legal system, and the digital world (Chu et al., 2022; Hopf et al., 2022; Iweins et al., 2013; Shpakou et al., 2021).

Ageism has major implications for our health and well-being (Chang et al., 2020). Research has shown that perceived exposure to age discrimination is associated with worse health and mental health status (Han & Richardson, 2015; Jackson et al., 2019). Consistently, self-directed ageism also results in reduced lifespan, worse health status, and poorer mental health outcomes (Chang et al., 2020; Levy, Slade, & Kasl, 2002; Levy, Slade, Kunkel, et al., 2002). The negative impact of ageism and, in particular, age discrimination also results in the exclusion of older people from the workforce and the social sphere (Noon & Ayalon, 2017; Stuckelberger et al., 2012; Walsh et al., 2017). Although much of the literature, to date, has focused on ageism toward older persons, ageism also affects younger people below the age of 50 (de la Fuente-Núñez et al., 2021). Nonetheless, its effects on younger populations have received less empirical support (de la Fuente-Núñez et al., 2021).

Despite its high prevalence and substantial impact, ageism is considered an understudied and underexplored topic (Ayalon & Tesch-Römer, 2018; Nelson, 2005). However, over the past decade, research on the topic has increased. Moreover, given the detrimental effects of ageism on older persons, the World Health Organization (WHO) launched a global campaign to combat ageism in 2016 with the goal of changing the way we feel, think, and act toward people because of their age to live in a world for all ages (Officer et al., 2016).

The present study aimed to examine variations in perceived age discrimination in the second half of life. Perceived age discrimination is different from objective age discrimination as it depends on respondents’ recognition, acknowledgment, and willingness to report the discriminatory event (Ayalon, 2016; Naff, 1995). This has been shown to be dependent on one’s mental health status so that more depressed individuals are more likely to report perceived exposure to age discrimination (Ayalon, 2016). In addition, familiarity with the term ageism also has been shown to be associated with the likelihood of reporting perceived age discrimination, with those individuals who are familiar with the term being more likely to report exposure to age discrimination (Okun & Ayalon, 2022). Nevertheless, perceived age discrimination is often used as an indicator of exposure to age discrimination because of its ease of use and because there are no agreed-upon objective criteria to determine exposure to age discrimination (Ayalon & Rothermund, 2018). This is not to say, however, that people’s subjective perceptions do not matter. In fact, there are substantial associations between perceived age discrimination and well-being, mental health, and health outcomes (Stokes & Moorman, 2019; Vogt Yuan, 2007), stressing the importance of subjective perceptions.

## Age, Period, and Cohort Effects

In the present study, we adopt a comprehensive approach that examines whether perceived age discrimination varies by age, period, or cohort. Age effects on perceived age discrimination are represented by variations in reports of perceived age discrimination by people of different age groups. Several studies have shown that younger people, rather than older people, are more likely to report perceived age discrimination (Ayalon, 2014; Ayalon & Gum, 2011; Gee et al., 2007; Vogt Yuan, 2007). This has been attributed to greater awareness of younger people of their rights and their greater willingness to call out unjust treatment. An analysis of reports of age discrimination by women in the context of the workforce has found a curvilinear effect, with perceived age discrimination being higher in those younger than 30 and over 50 (Gee et al., 2007). Nonetheless, there is research to show that compared with younger people, older persons are more likely to be viewed in a negative light (Kite et al., 2005). Moreover, during extreme times, older people are highly susceptible to ageist attitudes and behaviors (Ayalon, 2020).

Period effect, on the other hand, addresses the differential effects of the period in which data were collected on people’s reports of age discrimination. An example of a period effect could be the coinage of the term ageism by Robert Butler in 1969 (Butler, 1969). One would expect a sharp increase in reports of perceived age discrimination during that period, regardless of one’s age. Consistently, the introduction of the WHO campaign on ageism in 2016 (Officer et al., 2016) and the launching of the global report on ageism in 2021 (WHO, 2021) could mark the beginning of a new era, in which ageism receives international recognition and, as a result, people, regardless of their age, are more likely to acknowledge its occurrence. A historical analysis of 400 million words across 200 years has found increasing negativity of age stereotypes (Ng et al., 2015), thus, suggesting an increase in reports of perceived age discrimination over time.

Cohort effect refers to a situation in which people of a similar age experience a formative occurrence that affects their likelihood to report perceived age discrimination. For instance, it is expected that older people would report high levels of perceived exposure to age discrimination following the coronavirus 2019 (COVID-19) pandemic. This expectation is based on ample research demonstrating differential treatments of older persons during the pandemic, including explicit ageist statements and legislations in varied countries, worldwide (Previtali et al., 2020). Although younger people also were affected by the pandemic, their experiences are less likely to be attributed to ageism (Kauhanen et al., 2023). Another possible example of cohort effects concerns those cohorts that entered early adulthood during the Civil Rights era (Gee et al., 2007). Given the fact that political ideology often is shaped in young adulthood, it is possible that these individuals have become highly aware of discrimination and unequal treatment (Gee et al., 2007).

Teasing apart age, period, and cohort effects is challenging given the interdependence between the three (Bell & Jones, 2013). This is because cohort effects represent a combination of age and period. To our knowledge, only one study examined age, period, and cohort effects in the context of perceived age discrimination. The study, which examined women’s perceived age discrimination in the workforce, has found that age, rather than cohort or period, plays a major role in people’s reports of perceived age discrimination, with higher rates being reported in early adulthood (20s), dropping in the 30s and peaking in the 50s (Gee et al., 2007).

A better understanding of the role of age, period, and cohort effects in perceived age discrimination is important to the conceptualization of the etiology of age discrimination. If age effects appear to be most dominant, one can possibly argue for a more universal nature of perceived age discrimination, which is relatively unaffected by changing contextual events. This can assist in identifying those age groups most susceptible to perceived age discrimination that might benefit from further assistance and legal protection. On the other hand, the identification of period or cohort effects that results in peaks or drops in reports of perceived age discrimination can assist in the development of possible interventions to affect the report of perceived age discrimination. For instance, if we see an increase in reports following the introduction of new anti-ageist legislation, we can assume that this is due to increased awareness of the topic. Alternatively, a peak in reports of perceived age discrimination among older persons following the COVID-19 pandemic can stress the unplanned negative impact of the pandemic on certain groups in the population.

## Intersectionality

Ageism does not occur in a vacuum. It is often age in interaction with other attributes such as gender or ethnicity that result in different life experiences including the experience of discrimination (Farrell et al., 2022; Hopkins, 1980; Jones et al., 2017). For instance, the experience of an older White woman is quite different from the experiences of same-age Black or White men. Hence, it is important to examine the experience of perceived age discrimination from an intersectional perspective, which considers different attributes other than sole age. In the present study, we examine both gender and ethnicity as possible attributes that result in differential reports of perceived age discrimination.

There are several competing explanations of the intersection between age, gender, and ethnicity (Marcus & Fritzsche, 2015). One such explanation is the double or triple jeopardy theory (Taylor & Richards, 2019). This theory suggests that having multiple marginalizing attributes, such as older age, ethnic minority status, and female gender, result in worse outcomes compared with older men of the majority group. The rationale is that people experience cumulative disadvantages associated with their multiple marginal attributes, which put them at a greater risk. On the other hand, others have argued for the protective effects of having more than one marginalizing attribute, described as intersectional escape (Martin et al., 2019). In the case of older men versus older women, research has shown that the latter are seen as more likeable and less threatening in the workforce. Older men, in contrast, are expected to behave with lesser agency and to relinquish resources (Martin et al., 2019). Indeed, a different study that examined the experience of ageism among older male film directors has found that even though these were award-winning directors of high status, they experienced high levels of ageism when they reached older age (Lir & Ayalon, 2022).

The ethnic prominence theory suggests that numerical ethnic minority status overrides other attributes (Levin et al., 2002). This occurs both because discrimination toward ethnic minorities is more explicit and because individuals are more likely to think of themselves in terms of social group membership that are numerical minorities (Marcus & Fritzsche, 2015). Following this hypothesis, one would expect ethnic minorities to report higher levels of perceived age discrimination, regardless of their age. The subordinate male hypothesis, on the other hand, suggests that ethnic minority males are viewed most harshly because males are viewed as a source of conflict and threat and because of the salience of their ethnic minority status (Veenstra, 2013). Following this hypothesis, one would expect higher levels of perceived age discrimination reported by ethnic minority men.

## The Present Study

To date, research has been quite limited regarding the role of age, period, and cohort in shaping one’s reports of perceived age discrimination. Given the scarcity of research on the topic and the need to better understand the etiology of ageism (Marques et al., 2020), the present study offers a fresh look at the occurrence of perceived age discrimination in the second half of life. Given the limited research on the topic, we had no concrete hypotheses concerning the role of age, period, or cohort in shaping people’s reports of perceived age discrimination. As for the intersection of age with ethnic minority status and gender, given the fact that currently there is no consensus concerning the associations of these attributes with one’s experiences of age discrimination (Jones et al., 2017; Marcus & Fritzsche, 2015), we examine these attributes with no a priori hypotheses.

We expect our findings to provide important insights concerning the nature of perceived age discrimination over the life course from middle age to older age in different ethnic groups and across gender. These insights can inform policy by identifying those individuals most susceptible to report perceived age discrimination as well as specific periods or cohorts, which are characterized by particularly high or low levels of perceived age discrimination. This information, in return, can possibly guide contextual interventions.

Theoretically, our findings can inform theories on intersectionality by testing hypotheses concerning intersectional escape versus double jeopardy as well as the ethnic prominence theory. Our findings can possibly attest to the protective or alternatively harmful impact of multiple disadvantages, thus highlighting the role of intersectionality and the need to move away from examining age alone as a target of age discrimination. Given the limited research on the topic, we propose research questions rather than concrete hypotheses. The proposed research questions are addressed by the present study:

- What are the effects of age (lifespan) on perceived age discrimination?
- What are the effects of period (context) on perceived age discrimination?
- What are the effects of cohort (generation) on perceived age discrimination?
- Do age–period–cohort (APC) effects vary across gender and/or ethnicity?

## Method

### The Sample

The Health and Retirement Survey (HRS) is a longitudinal representative panel study of U.S. citizens over the age of 50 that is publicly available for use. Data collection started in 1992 and occurs on a biennial schedule. The HRS oversamples Blacks, Hispanics, and residents of Florida. A new cohort of individuals between the ages of 51 and 56 is added every 6 years. As of 2006, half the sample is interviewed in person and the remaining half is interviewed by phone. Starting in early 2003, a small subset of interviews was conducted online. A self-report psychosocial survey (leave-behind questionnaire) was introduced in 2004 with longitudinal data on the survey being collected every 4 years. The study is funded through a cooperative agreement between the National Institute of Aging and the University of Michigan (U01 AG009740) with supplemental funding from the U.S. Social Security Administration (Fisher & Ryan, 2017). The HRS data collection was approved by the Institutional Review Board of the University of Michigan.

### Measures

Age, gender, and ethnicity (White, Black, Latino, other) were gathered based on self-report. Perceived exposure to age discrimination was constructed based on a six-item scale to assess everyday discrimination (the sixth item was added in 2008; “you receive poorer services or treatment than other people from doctors or hospitals”). Response options range on a 6-point scale between never and every day (Rogers et al., 2015; Sutin et al., 2016; Williams et al., 2003). Cronbach alpha ≥ 0.8 across waves. Those who indicated exposure were asked to attribute the exposure to varied reasons including age (Kessler et al., 1999). In the present study, those who attributed their exposure to age were considered as reporting perceived exposure to age discrimination, whereas all other respondents who participated in the survey and did not attribute the exposure to age or did not report exposure to everyday discrimination were considered as not reporting perceived age discrimination.

### Data Preparation

In the present study, we relied on seven waves of data collected between 2006 and 2018. We merged observations taken on two consecutive years, so that observations taken in 2006 and 2008 were coded as representing 2007. The overall sample size consisted of 49,302 observations (14,587 in 2006–2008; 15,485 in 2010–2012; 13,636 in 2014–2016; 5,509 in 2018). Of these, 29,226 were women and 20,024 men. A small part of the observations did not provide an answer regarding perceived discrimination and were removed from the final analysis (resulting in 26,442 women and 18,609 men).

The decision to merge two consecutive waves when possible was led by the otherwise relatively small sample size of some ethnic groups that would have been unsuitable for an APC analysis. This strategy has been used in the past, see Bramajo (2022). This process was followed for all waves except for 2018, which was not combined with 2020. Given the effects of the COVID-19 pandemic, it was inadequate to combine the 2020 wave with previous ones. A second assumption was to consider that the prevalence in 2018 was similar to that reported in 2019 in order to have symmetric 4-year intervals. Age was categorized using a 4-year bracket (e.g., 50–53, coded as 52 because age is a continuous variable in the analytic strategy). Hence, we analyzed data from four periods and 15 age groups, separated by gender (men/women) and ethnic minority status (White, Black, Latino, other). More details about the composition of the sample and the APC tabulation can be found in Supplementary Tables 1A and 2A.

### Analysis

First, we explored the data by estimating the age-standardized incidence (by using the proportion of age-specific population over all the waves as the standard structure) to inspect the first trend of incidence of perceived age discrimination. Next, we relied on a set of APC models to determine the separate effects of aging (age) factors, contextual (period) factors, and generational (cohort) factors in perceived age discrimination. APC models represent a descriptive device that can partially determine the effects separately. Due to the identification problem (a perfect collinearity between the three dimensions of age, period, and cohort), we had to resort into a particular strategy to successfully model any outcome variable because of the potential infinite solutions. It is important to note that there is a variety of ways in which APC models can be conducted. No particular method is perfect, and all methods have certain limitations (Bell & Jones, 2014; Carstensen, 2007; Holford, 1992; Yang et al., 2008). We followed the approach suggested by Carstensen (2007), which considers age, period, and cohort as continuous dimensions and could be an extension of the method of constrained dimensions in order to have estimable functions (Holford, 1983).

To avoid perfect collinearity, this method constrains (one or two) dimensions (generally period or cohort), by making the average trend effect equal to 0 and presenting risk ratios compared to the average trend. These effects, known as nonlinear, second-order, or curve effects, are fully identifiable and independent of the chosen parameterization. This method also assumes that the linear drift (the linear change) is attributed to the nonconstrained dimension and presents risk ratios to a selected reference category. This assumption, mathematically arbitrary, is largely driven by theory. Age is expressed in age-specific rates, depending on a reference period or cohort, generally by the choice of the user. Because we do not have a priori any theoretical insights telling us how the linear effects should behave in this case, we opted to constrain both period and cohort dimensions, getting second-order effects that are fully identifiable, only focusing on identifying nonlinear effects that are fully identifiable, and getting cross-sectional age effects (based on the reference period, 2007; e.g., 2006–2008 data collection, combined). These sets of flexible APC models are available in the Epi Package in R free software (EpiCarstensen et al., 2021).

The model we chose is the Ad-P-C parameterization. The basic formula for the chosen model is the following: ln(*d*(*a*, *p*)) = *r*_pe_(*a*) + δ + *g*(*p*) + *h*(*c*). In this model, the log rates of age discrimination (*d*) on a specific age group (*a*) for the chosen reference period (*p*)—in this case 2007—are essentially the sum of the estimable functions of age-specific rates. Both the curve period effects *g*(*p*) and the curve cohort effects *h*(*c*) are estimable functions. And we separately estimate the linear drift δ, not allocated a priori to either the cohort or period dimension (and ultimately, as the results show, allocating the drift to a particular dimension would have not made any meaningful difference).

## Results

**Figure 1.**
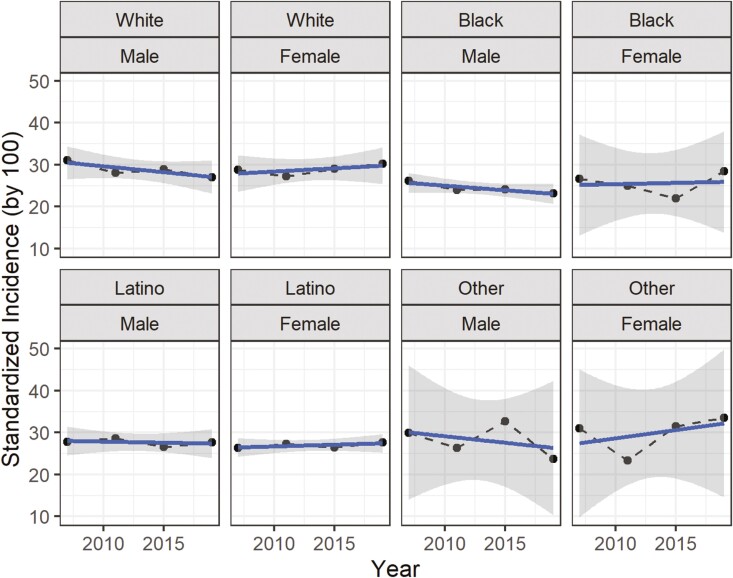
Age-standardized incidence (in %) by year, gender, and ethnicity.

Figure 1 presents the age-standardized incidence over time across all waves, separately by gender and ethnicity. Regardless of gender or ethnicity, the incidence of perceived age discrimination remains relatively stable, ranging between 25% and 30% on average. The lack of change is consistent with Table 1, which presents the maximum-likelihood values for linear drifts over time. Basically, we cannot establish any linear deviation (with values that can be significantly determined as being above or below the average threshold of 1). That said, it is certainly possible that nonlinear effects are present in the model. The lack of a strong linear drift also confirms that allocating the linear effects to either period or cohort is relatively irrelevant in this case.

**Table 1.** Values of Average Maximum-Likelihood Drifts for Linear Change

Gender	Ethnicity	Expected value (to the average trend)	2.5%	97.5%
Men	White	0.995	0.987	1.003
	Black	0.987	0.965	1.009
	Latino	1.004	0.985	1.024
	Other	0.984	0.947	1.023
	All	0.996	0.982	1.009
Women	White	1.005	0.998	1.012
	Black	0.993	0.974	1.012
	Latino	1.005	0.991	1.020
	Other	1.012	0.980	1.045
	All	1.002	0.991	1.013

**Figure 2.**
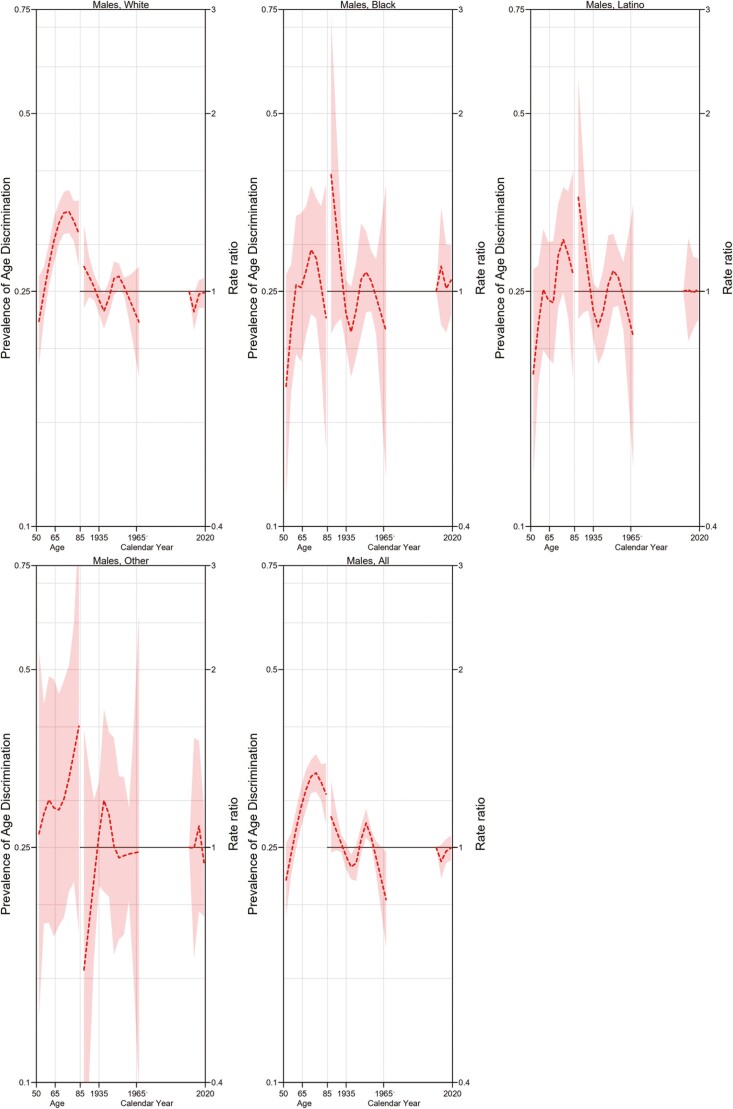
Age–period–cohort model for age-perceived discrimination for women by ethnicity.

**Figure 3.**
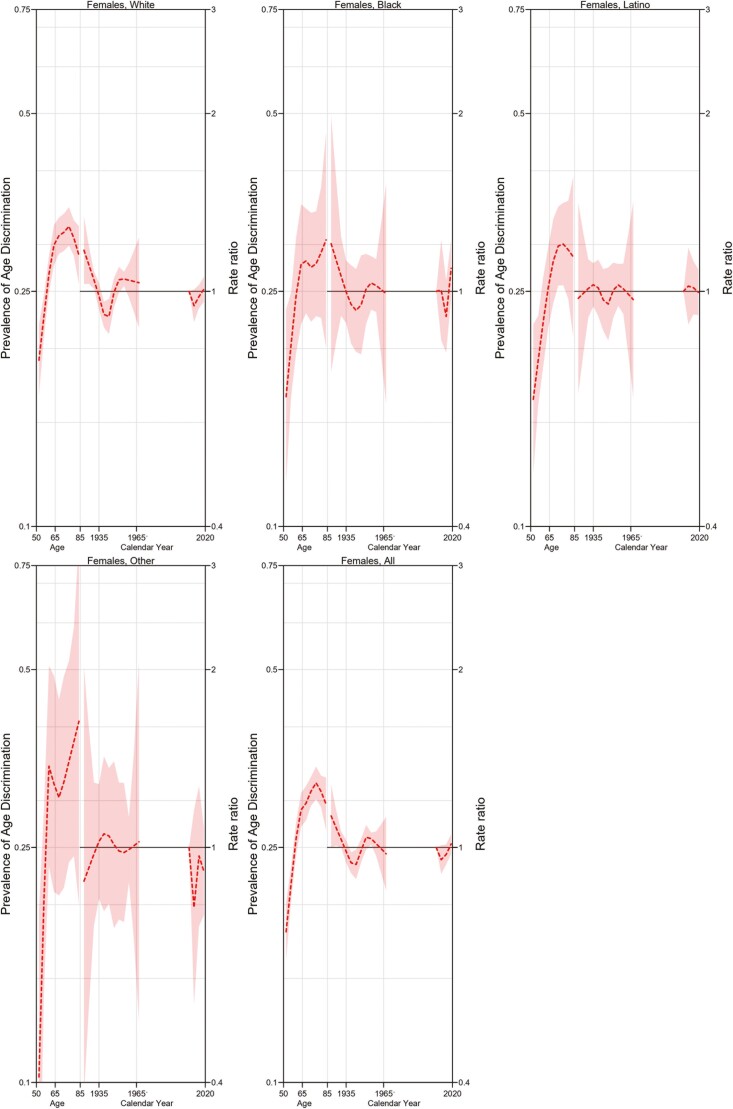
Age–period–cohort model for age-perceived discrimination for men by ethnicity.

Figures 2 and 3 demonstrate the APC model for women overall and divided by ethnic group affiliation and for men overall and divided by ethnic group affiliation. For both women and men, our findings show that perceived age discrimination increases with age but reaches a plateau around the age of 75. This pattern appeared consistent regardless of ethnic group affiliation. Both men and women born in the 1940s show a slight decrease in perceived age discrimination. Those born in the early 1960s, on the other hand, show a slight increase in perceived age discrimination. In addition, White women born prior to 1935 show a slight but significant increase in perceived age discrimination when compared to the overall trend. It is important to note, however, that the shape of cohort effects appears similar for Blacks or Latinos and comparable to the overall population, where cohort effects seem to be significant arguably due to a larger sample size. No other remarkable cohort or period effects were noted.

## Discussion

Ageism in general and perceived age discrimination in particular have received considerable attention in recent years (Ayalon & Tesch-Römer, 2018; Robinson-Lane et al., 2022), not only as research topics, but also as real-life issues that can compromise people’s well-being and quality of life (WHO, 2021). The present study, for the first time, adapted an analytic framework to possibly distinguish between age, cohort, and period effects. This distinction is important because it can provide important insights concerning the etiology of perceived age discrimination and ways to overcome it. The study also examined intersectionality in relation to perceived age discrimination, thus evaluating different theoretical explanations concerning the protective versus harmful effects of intersectionality.

The most notable finding of the present study concerns the consistent age effect, regardless of gender or ethnicity. Our findings show that the likelihood of reporting perceived age discrimination increases with age and subsides around the age of 75. Although several studies have found that it is younger people who are more likely to report perceived age discrimination (Vogt Yuan, 2007), research has focused more extensively on the discrimination of older persons on the basis of age (Ayalon & Tesch-Römer, 2018). Moreover, the scientific basis for the impact of ageism or age discrimination on older persons’ quality of life, well-being, and health is substantially stronger than current evidence concerning younger persons (de la Fuente-Núñez et al., 2021).

The relative plateau in terms of perceived age discrimination reached at around the age of 75 could possibly reflect an “on time event.” As already noted, there is a clear difference between perceived age discrimination and objective age discrimination with the former requiring the respondent to notice the event, interpret it as discriminatory, and report it as such (Ayalon, 2016; Naff, 1995). The stereotype embodiment theory suggests that people internalize negative age stereotypes throughout their lives and in old age, when these stereotypes become self-relevant, they are directed toward oneself (Levy, 2009). Hence, it is possible that the older age groups (75+) have already internalized negative age stereotypes and as a result are less likely to acknowledge age discrimination and view it as an unacceptable phenomenon.

We also found that the cohorts born in the 1940s had a slight decrease in perceived age discrimination when compared to the average trend, both for men and women. Furthermore, cohorts of both men and women born in the early 1960s also perceived a higher age discrimination when compared to the others. This finding as well could be explained by the subjective nature of perceived age discrimination. It is possible that compared with those born in the 1960s, those born in the 1940s, almost 30 years before the term ageism was coined, grew up being less aware of age discrimination and therefore are less likely to report being exposed to it compared with those who grew up when the term was already in use. This, however, stands in contrast with the finding that White women born before 1935 showed a slight but significant increase in perceived age discrimination. Because both the increase and decrease in perceived age discrimination in these age cohorts appear minimal, it is unclear what the meaning of these findings is, which could be attributed to the sample size.

In contrast to past research and theory that have highlighted the importance of intersectionality, our findings do not provide evidence concerning its effects. Hence, we do not provide support for the theory of intersectional escape (Martin et al., 2019), nor to the double jeopardy (Taylor & Richards, 2019), or the ethnic prominence theory (Levin et al., 2002). Instead, our findings suggest that it is chronological age that makes older persons more susceptible to perceived age discrimination, rather than other attributes such as ethnicity or gender. This finding is consistent with the majority of past research, which has not paid substantial attention to different grounds of marginalization in the understanding of ageism (Krekula et al., 2018).

The present study aimed to obtain insights concerning the etiology of perceived age discrimination by identifying certain periods or periods in interaction with age (i.e., cohorts) that are more susceptible to report age discrimination. However, our findings suggest that it is mainly age effects rather than period or cohort effects that contribute to perceived age discrimination. There are several plausible mechanisms that could be responsible for the increase in perceived age discrimination that comes with age. One such mechanism could be societal attitudes toward older persons and in particular toward visible signs of aging. It is possible that as people age and demonstrate more visible signs of aging, they also are more likely to experience discrimination. Another option would be that older persons are more tuned to discriminatory events compared with younger people. However, this hypothesis has largely been refuted in past research, which has argued that older persons are more tuned toward registering and recalling positive events (Löckenhoff & Carstensen, 2004). Regardless of the exact mechanism responsible for the increase in reports of perceived age discrimination with age, our findings show that age effects are quite constant over time and have not been affected by major societal events that took place over time nor by gender or ethnicity. As such, interventions could possibly be quite standardized and geared toward reducing ageism towards older persons, regardless of their other attributes such as gender or ethnicity. The fact that after the age of 75, there was a plateau in reports of perceived age discrimination could highlight a need to increase awareness of ageism among older persons at the age of 75 and older. This is because past research has shown a link between increased awareness of the topic and increased willingness to report exposure to age discrimination (Okun & Ayalon, 2022).

The present findings should be viewed considering their limitations. First, although this is a large representative survey of people over the age of 50, data collection of perceived age discrimination started only in 2006. Hence, our analysis was limited to only four periods. In addition, we examined perceived age discrimination only in the second half of life, thus, we were not able to have a life-course perspective on the topic. Our findings show that when it comes to changes in perceived age discrimination, it is mainly chronological age that matters, with both men and women, regardless of their ethnic origin, being more likely to report perceived age discrimination as they grow older, until they reach the age of 75, when a plateau in the reports of perceived age discrimination is reached. However, it is important to note that our sample was representative only of the U.S. population. Clearly, there are notable variations in the manifestations of age discrimination across countries and regions (Ayalon & Roy, 2022). Hence, it is possible that APC effects manifest differently in different countries and cultural contexts. Also, given the sampling design of the HRS, all residents of the same household were interviewed. This could potentially result in some bias due to nonindependence of observations. Nevertheless, this sampling mechanism is considered to represent the U.S. citizens over the age of 50. Moreover, past research that has conducted APC analysis used the entire HRS data set as well, while not accounting for nonindependence among respondents, given the otherwise reduced sample size (Hawkley et al., 2019). Last, although we found some cohort effects, these effects appear to be quite minimal, thus their significance is unclear and they could possibly reflect the large sample size, rather than meaningful differences.

To sum up, our findings indicate that as people grow older, they are more likely to report perceived age discrimination at least up to the age of 75. Thus, regardless of ethnicity or gender, and unrelated to possible cohort or period effects, it is older persons who are most susceptible to perceived age discrimination. This group of older persons requires further attention and should be the target of future interventions aimed to reduce or prevent age discrimination. Specifically, age discrimination laws and policies should protect this age group. In addition, educational interventions and social contact interventions, which have shown to reduce ageist stereotypes (Burnes et al., 2019), can specifically address the experiences of older age groups given the greater likelihood of reports of perceived age discrimination in these groups. More research is needed to further understand the mechanism that links between age and perceived age discrimination. If visible signs of aging are responsible for societal discrimination of older persons, educational interventions should address stereotypes of older persons’ appearance as a means to reduce societal discrimination. However, if older persons (up to the age of 75) are more tuned to discrimination compared with younger persons, and this is what explains the higher levels of perceived age discrimination reported by them, interventions need to focus on older persons’ interpretation of social interactions. As people over the age of 75 reached a plateau in their reports of age discrimination, this group in particular could benefit from further education about ageism. Our findings also point to limited variability in older persons’ reports of age discrimination, thus, interventions may not necessarily have to address gender or ethnicity when targeting perceived age discrimination.

## Supplementary Material

igad094_suppl_Supplementary_Material
