# Supplementary material for: Perceived Age Discrimination in the Second Half of Life: An Examination of Age, Period, and Cohort Effects
Source: Innov Aging. 2023 Aug 30;7(8):igad094. doi: 10.1093/geroni/igad094 (PMC11025380; doi:10.1093/geroni/igad094)
Supplement: igad094_suppl_Supplementary_Material [file igad094_suppl_supplementary_material.docx]

**Online Supplementary Material**

Table 1A: Reported cases by sex and ethnicity during the 2007-19 period

| Period | Sex | Ethnicity | Cases | Observations |
| --- | --- | --- | --- | --- |
| 2006-08 (2007) | Women | White | 1707 | 5828 |
| 2006-08 (2007) | Men | White | 1378 | 4383 |
| 2010-12 (2011) | Women | White | 1552 | 5580 |
| 2010-12 (2011) | Men | White | 1186 | 4234 |
| 2014-16 (2015) | Women | White | 1406 | 4717 |
| 2014-16 (2015) | Men | White | 1014 | 3410 |
| 2018 (2019) | Women | White | 602 | 1911 |
| 2018 (2019) | Men | White | 408 | 1399 |
| Total 2007-19 | Total | White | 9253 | 31462 |
| 2006-08 (2007) | Women | Black | 173 | 672 |
| 2006-08 (2007) | Men | Black | 115 | 439 |
| 2010-12 (2011) | Women | Black | 215 | 895 |
| 2010-12 (2011) | Men | Black | 170 | 638 |
| 2014-16 (2015) | Women | Black | 203 | 940 |
| 2014-16 (2015) | Men | Black | 160 | 677 |
| 2018 (2019) | Women | Black | 103 | 406 |
| 2018 (2019) | Men | Black | 66 | 285 |
| Total 2007-19 | Total | Black | 1205 | 4952 |
| 2006-08 (2007) | Women | Latino | 287 | 1102 |
| 2006-08 (2007) | Men | Latino | 159 | 601 |
| 2010-12 (2011) | Women | Latino | 379 | 1504 |
| 2010-12 (2011) | Men | Latino | 214 | 809 |
| 2014-16 (2015) | Women | Latino | 383 | 1449 |
| 2014-16 (2015) | Men | Latino | 217 | 807 |
| 2018 (2019) | Women | Latino | 162 | 610 |
| 2018 (2019) | Men | Latino | 93 | 337 |
| Total 2007-19 | Total | Latino | 1894 | 7219 |
| 2006-08 (2007) | Women | Other | 51 | 174 |
| 2006-08 (2007) | Men | Other | 37 | 122 |
| 2010-12 (2011) | Women | Other | 55 | 238 |
| 2010-12 (2011) | Men | Other | 49 | 177 |
| 2014-16 (2015) | Women | Other | 79 | 269 |
| 2014-16 (2015) | Men | Other | 54 | 193 |
| 2018 (2019) | Women | Other | 41 | 147 |
| 2018 (2019) | Men | Other | 22 | 98 |
| Total 2007-19 | Total | Other | 388 | 1418 |

Table 2A: Age-Period-Cohort tabulation in middle points.

| Age Groups | MidYear | 2007 | 2011 | 2015 | 2019 |
| --- | --- | --- | --- | --- | --- |
|  | Midpoint | Birth Cohort (Mid Point) | | | |
| 50-53 | 52 | 1955 | 1959 | 1963 | 1967 |
| 54-57 | 56 | 1951 | 1955 | 1959 | 1963 |
| 58-61 | 60 | 1947 | 1951 | 1955 | 1959 |
| 62-65 | 64 | 1943 | 1947 | 1951 | 1955 |
| 66-69 | 68 | 1939 | 1943 | 1947 | 1951 |
| 70-73 | 72 | 1935 | 1939 | 1943 | 1947 |
| 74-77 | 76 | 1931 | 1935 | 1939 | 1943 |
| 78-81 | 80 | 1927 | 1931 | 1935 | 1939 |
| 82+ | 84 | 1923 | 1927 | 1931 | 1935 |
